# Instructors’ expressive nonverbal behavior hinders learning when learners’ prior knowledge is low

**DOI:** 10.3389/fpsyg.2022.810451

**Published:** 2022-08-19

**Authors:** Mengke Wang, Zengzhao Chen, Yawen Shi, Zhuo Wang, Chengguan Xiang

**Affiliations:** Faculty of Artificial Intelligence in Education, Central China Normal University, Wuhan, China

**Keywords:** nonverbal behavior, learning performance, affective experience, video lectures, multimedia learning, nonverbal expressiveness, prior knowledge

## Abstract

This study investigated the influence of instructors’ expressive nonverbal behavior and nonexpressive nonverbal behavior in video lectures on students’ learning performance and affective experience. We conducted two rounds of experiments using the same materials and procedures, the only difference being the participants. In each round of experiments, participants were randomly assigned to expressive condition or nonexpressive condition. 227 rural primary school sixth-graders took part in experiment 1, participants in expressive condition had better affective experiences and perceived tasks as less difficult, but had lower learning performance than participants in nonexpressive condition. 175 sixth-graders from urban primary schools participated in experiment 2. The results showed that instructors’ expressive nonverbal behavior also improved students’ affective experience and reduced students’ perception of task difficulty, but there was no significant difference in learning performance between the two groups. Comparing the pretest scores of students in the two experiments, it was found that the pretest scores of participants in experiment 2 were higher than those in experiment 1. Overall, instructors’ expressive nonverbal behavior can improve students’ affective experience and reduce their perception of task difficulty. However, when students’ prior knowledge is relatively low, instructors’ expressive nonverbal behavior hinders students’ learning performance. We suggest that teachers adopt expressive nonverbal behavior when lecturing because it is beneficial to maintain students’ long-term interest in learning. However, it should be noted that the difficulty of learning material should be determined by students’ prior knowledge.

## Introduction

Under the global COVID-19 pandemic, video lectures become more widely used in school education than ever (Gouëdard et al., 2020; Wang et al., 2021). In the video lectures with instructor presence, the eye gaze, facial expression, gestures, and paralinguistic speech characteristics of instructors could have different degrees of expression. For example, some instructors smile in the video, use pointing gestures to guide learners’ attention, and speak in cadence. While there are also some instructors who have no facial expressions, do not use gestures, and speak in a single tone. The former instructors used expressive nonverbal behaviors, while the latter instructors used nonexpressive nonverbal behaviors. Nonverbal expressiveness describes the nonverbal behaviors with different levels of expression and is conceptualized as the manifestation of those nonverbal behaviors which communicate animation, enthusiasm, interest, and overall expressiveness (Roter et al., 2006). Instructors with expressive nonverbal behaviors are often considered as warm, intimate, and approachable, while instructors with nonexpressive nonverbal behaviors are often considered as cold, remote, and unapproachable.

Instructors’ nonverbal expressiveness affects learners’ emotions, motivation, interest, and affective experience. Learners who learn from instructors with expressive nonverbal behavior tend to report more positive emotional experiences. However, liking does not always lead to learning (Wilson et al., 2018). According to previous studies on teaching videos with instructors’ presence, although teaching videos with instructors present were more favored by learners than those with texts or audio alone, learners also believed that they paid more attention when watching videos with instructors present. However, the results of comprehension tests showed that videos of instructors present did not improve learners’ scores on comprehension tests, and learners paid more attention to the teacher than to the material. In addition, other studies have shown that instructors’ visual cues and motor characteristics also attract learners’ attention, depleting attention resources that should be used to process learning material (Kizilcec et al., 2014). Compared with nonexpressive nonverbal behaviors, expressive nonverbal behaviors of instructors have more motor characteristics. Therefore, empirical research is needed to confirm whether expressive nonverbal behaviors of instructors can distract learners’ attention and decrease their learning performance.

Furthermore, studies in multimedia learning mainly focused on adult learners (e.g., Stull et al., 2018; Beege et al., 2020; Horovitz and Mayer, 2021) and the impact of video lectures on younger learners have not been fully studied. Compared to adult learners, younger learners, especially primary learners, have relatively little knowledge and shorter attention spans. Thus, it is more difficult for them to establish connections with new knowledge from self-directed video learning. These difficulties may lead to increased cognitive load and boredom emotion. More research is needed to investigate the influence of video lectures on younger learners’ learning performance and affective experience. Research on this issue will facilitate video design for younger learners. Therefore, this study aims to explore the influence of instructors’ nonverbal expressiveness on primary school learners’ learning performance and affective experience (including learning experience and learning satisfaction).

## Literature review

In empirical studies, researchers have linked instructors’ nonverbal behavior with classroom outcomes, forming varying labels such as enthusiasm (Brophy and Good, 1986; Marsh, 1994; Jackson et al., 1999; Patrick et al., 2003; Kunter et al., 2008, 2011), immediacy (Mehrabian, 1969; Andersen, 1979; Velez and Cano, 2008; Liu, 2021), and communication style (Norton, 1978; Sallinen-Kuparinen, 1992). Although the frameworks of these terms mentioned above are not identical, they all share a common variable, namely nonverbal expressiveness. Its core is the application of nonverbal behavior. Expressive instructors are those very expressive in their faces, hands, voices, and body orientation toward their audience. Normally, they make use of demonstrative gestures, vivid facial expressions, meaningful body movements, and varying voice tones (Keller et al., 2016). While nonexpressive instructors are those who have not mobilized nonverbal behavior. They present an emotionless facial expression, avoid eye contact, and rarely employ gestures, their intonation does not change and body posture remains stiff.

### The positive impact of instructors’ expressive nonverbal behavior on learning

It seems to be an intuitive common sense that instructors who smile, gesticulate, and express enthusiasm are viewed by students as being more student-oriented, organized, stimulating, and knowledgeable and as having more rapport than nonexpressive instructors. A multitude of studies confirmed that instructors’ expressive behavior contributes to preventing boredom, increasing interest, improving satisfaction, and motivating students to pursue their studies by continuous shifts in the various channels of their nonverbal behavior (Liu, 2021; Moè et al., 2021).

Emotional response theory and emotional contagion theory both provide valuable insight into how instructors’ expressive nonverbal behavior promotes students’ learning. According to the emotional response theory, instructors’ expressive nonverbal behavior will induce an emotional response in learners (McCroskey et al., 2006; Mottet and Beebe, 2006). When an instructor shows a direct gaze and a happy face, it may prime students’ positive response and interpret it as ‘the instructor is happy to teach me.’ Once an instructor’s nonverbal behavior stimulates students’ emotional responses, it could further motivate students to engage in activities and facilitate learning (Mayer, 2014; Liew et al., 2017; Pi et al., 2020). Besides, smiling expressions, open body posture, and passionate tone, all suggest a high valence of positive emotion which could affect learners’ emotions through the process of emotional contagion (Pekrun et al., 2002). Becker et al. (2014) believed that emotions conveyed through instructors’ nonverbal behavior are as important as the knowledge itself.

What’s more, studies found that without external incentives audiences are more strongly influenced by delivery style than by content. Instructors’ nonverbal cues could affect students’ judgment of instructors’ professional ability. Smiles, positive voice tone, head nods, eye contact, and gestures could encourage students’ positive attitudes (Goldberg and Mayerberg, 1973; Kaufman 1975, unpublished doctoral dissertation^1^; Woolfolk et al., 1977; Chaikin et al., 1978; Guyer et al., 2019; Lawson et al., 2021a). When instructor expressiveness overrides the effect of lecture content on student evaluations, the “Dr. Fox Effect” happens, this is also known as the educational seduction phenomenon. Students’ evaluation of instructors’ professional ability, in turn, affects students’ cognitive engagement, and ultimately affects the learning result. Although “Dr. Fox Effect” causes researchers’ concern about the validity of students’ evaluation of teaching, it is sufficient to illustrate the importance of instructors’ nonverbal expressiveness in teaching (Peer and Babad, 2014).

### The negative impact of instructors’ expressive nonverbal behavior on learning

Although instructors’ expressive nonverbal behavior enhances students’ emotional experience, they do not definitely promote students’ cognitive performance. Andersen and Withrow (1981) recorded three different levels of nonverbal expressiveness videotapes to different audiences and found that nonverbal expressiveness predicted 22% of the variance in students’ affect learning, but it was not a significant predictor for behavioral commitment or cognitive learning. Chaikin et al. (1978) conducted an exploratory study, on the condition that the instructor behaved with eye contact, leaning forward, smiling, and head nods produced more positive ratings from fifth-grade students than on the other condition that the instructor behaved with little eye contact, leaning away, frowning, and shake head. But there was no significance in learning performance between the two conditions. What’s more, some studies have documented the negative effect of instructors’ expressive nonverbal behavior on students’ learning outcomes. Basow and Distenfeld (1985) claimed that although the expressive instructor received the highest student evaluations, students who watched a nonexpressive female instructor had the highest achievement. Besides, McKinney et al. (1984) reported that instructors’ high level of expressive nonverbal behavior increases classroom management problems in primary school.

Expressive nonverbal behavior contains more dynamic elements (e.g., making gestures, walking, showing different facial expressions) than nonexpressive nonverbal behavior. This dynamic information attracts students’ attention (Shaddy and Colombo, 2010). The limited-capacity assumption reminds us that each channel in the human information processing system has a limited capacity. Instructors’ nonverbal behavior is needed to be processed and integrated with the learning materials. They may compete with the learning materials for limited capacity, thereby affecting the processing of learning materials. As Ayres and Sweller (2005) demonstrated when learning from video lectures presenting an instructor and learning materials, students are required to integrate disparate information, and they have to split their attention to process information from more than one source. Instructors’ nonverbal behavior may add extraneous cognitive load on students, which imposes an additional processing burden on a learner’s mind (extra processing in the brain) and negatively affects cognitive outcomes.

### The present study

The current study aimed to explore the influence of instructors’ different levels of nonverbal expressiveness in video lectures on elementary students’ learning performance and affective experience (including learning experience and learning satisfaction). We conducted two experiments with the same experimental materials and procedures but different samples. In experiment 1, participants were from a rural primary school. In experiment 2, participants were from an urban primary school.

## Experiment 1

### Research questions and hypothesis

Based on emotional response theory and emotional contagion theory, the expressive instructor may improve students’ affective experience (including learning experience and learning satisfaction) more than the nonexpressive instructor. Thus, we formulated the following hypothesis:

*H1*: Compared with the nonexpressive condition, students who learn from the instructor with expressive nonverbal behavior will have a better learning experience.

*H2*: Compared with the nonexpressive condition, students who learn from the instructor with expressive nonverbal behavior will report higher learning satisfaction.

As for learning performance, existing studies presented contradictory results. On the one hand, instructors’ expressive nonverbal behavior has advantages in maintaining students’ interest and motivation in learning, leading to high learning performance. On the other hand, instructors’ expressive nonverbal behavior competes with learning materials for students’ attention and limited working memory, resulting in poor learning performance. Therefore, our research question was: What is the effect of instructors’ nonverbal expressiveness on students’ learning performance?

### Method

#### Participants and design

Participants in the present study were students from a township elementary school located in Guizhou, China. A total of 227 students in sixth grade responded to the survey. The sample consisted of 114 males and 113 females. All participants were randomly assigned to two groups: 115 (male = 54, female = 61)students grouped in the expressive condition and watched the video lecture with an expressive instructor. 112 (male = 60, female = 52) students grouped in the nonexpressive condition and watched video lectures with a nonexpressive instructor. The data were collected through questionnaires that were administered in the school classes by a research assistant and six school instructors. The study protocol was approved by the ethics committee of Central China Normal University.

#### Material

We created two learning videos with different nonverbal expressiveness. To reduce the influence of the emotional attributes of the learning content on students’ emotions, the classification of vertebrates was chosen as teaching content. Within the videos, a female on-screen instructor was implemented, while the learning content was presented via slides. The authors prepared a list of body movements, facial expressions, and tone of voice, which should be performed in the respective condition. Before the videos were recorded, the instructor received concrete instructions from the authors of this study. The instructor was trained to perform targeted nonverbal behavior as flawlessly as possible before the final videos were recorded.

The illustration of the experimental manipulation is presented in Table 1. In the expressive condition, the instructor smiled, looked directly at the camera and her body faced the camera. When she pointed to the teaching material, her eyes were directed towards the teaching material and her body was sideways towards the teaching material. She used a positive tone when lecturing and varied the voice speed and intonation with the content. The learning time was limited to 522 s.

**Figure 1:**
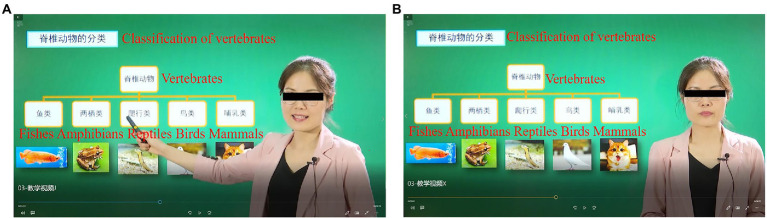
Screenshots of the two versions in the experiment (after privacy treatment). **(A)** The expressive version and **(B)** the nonexpressive version.

**Figure 2:**
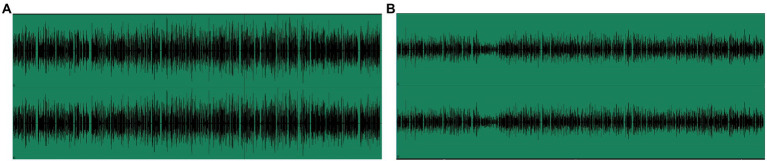
Waveforms of the two versions in the experiment. **(A)** The expressive version and **(B)** the nonexpressive version.

In the nonexpressive condition, the instructor’s face was serious. She looked down or toward the camera, and her body was facing forward. She did do not use hand gestures or change body orientation. She used a monotone tone in the whole lecture. The learning time was limited to 455 s. Screenshots are shown in Figure 1, and the waveforms are shown in Figure 2. Taking into account the ecological validity of the experiment, both experimental treatments were relatively mild and not very exaggerated.

**Table 1:** Illustration of the experimental manipulation.

Nonverbal behavior	Expressive condition (behavior, second)	Nonexpressive condition (behavior, second)
Facial expression	Smile, 51 s	No facial expression all the time
Eye gaze	Direct gaze(looked straight to the camera), 371 s	Direct gaze (looked straight to the camera), 93 s
	Guided gaze (used guided gaze to direct attention to the slides), 151 s	Averted gaze (looked down), 362 s
Gesture	Pointing gestures (pointed to the slides), 268 s	No gestures all the time
Body orientation	Frontal body,157 s;lateral body, 365 s	Frontal body all the time
Voice tone	Positive voice tone, voice speed and intonation change with the content	Monotone all the time

#### Measurements

##### Prior knowledge test

The prior knowledge test was an 8.5 × 11 inch sheet of paper that asked participants to provide their gender and class. This test also included (1) three true or false items(e.g., Snails are not vertebrates,1 point each, 3 points in total), (2) two single-choice items (e.g., Which reptiles are listed? (A) gecko; (B) frog; (C) earthworm; and (D) snail) in which every item had four choices and only one correct answer (1 point each, 2 points in total), and (3) one multiple-choice question (Which of the following descriptions of the characteristics of the tortoise are correct? (A) Turtles can live in water or on land, so they are amphibians. (B) The tortoise is a vertebrate. (C) The tortoise is a reptile. Two points). All the items in the test were developed by the researchers and examined by a biology professor to ensure expert validity. The total possible score was 7 points. The higher the score on this test indicated a higher degree of prior knowledge.

##### Learning performance test

Under the guidance of a biology professor, the researcher who was familiar with the learning content developed the learning performance test. It included (1) five fill-in-blank items (e.g., The definition of a vertebrate is _____.); (2) three true or false items, (e.g., Birds are warm-blooded); and (3) four short-answer items (e.g., Which category of vertebrates does the tortoise belong to?). The total possible score was 18 points. The higher the score indicated a higher degree of learning performance.

##### Learning experience questionnaire

The learning experience questionnaire (Stull et al., 2018) was used to measure participants’ learning experience for the two video lectures. Students were asked to rate their experience on the following 9 items: “I felt that the subject matter was difficult,” which was reverse coded in the analysis, and “I enjoyed learning this way,” “I would like to learn this way in the future,” “I feel like I have a good understanding of the material,” “After this lesson, I would be interested in learning more about the material,” “I found the lesson to be useful to me,” “I felt like the instructor was working with me to help me understand the material,” “I found the instructor’s teaching style engaging,” and “I felt motivated to try to understand the material.” The items could be rated on a 7-point Likert scale ranging from 1 (“strongly disagree”) to 7 (“strongly agree”). In the current study, Cronbach’s alpha for the learning experience questionnaire was 0.822.

##### Learning satisfaction questionnaire

This questionnaire was based on the Video Course Learning Satisfaction Questionnaire. The original 20 items scale was modified such that items that did not fit the context of the current study were deleted (e.g., items about instructor-student interaction). The modified scale had three 5-point Likert-scale items (1 = absolutely disagree, 5 = absolutely agree): instructor teaching had one item, teaching content had one item, and learning environment and equipment had one item. Higher scores indicated higher learning satisfaction. Cronbach’s alpha for the learning satisfaction questionnaire was 0.786.

#### Procedure

The study was carried out in six multimedia classrooms, with six naturally occurring classes. Each condition was randomly assigned to three classes. 115 students (male = 54, female = 61)watched the instructor with expressive nonverbal behavior video lecture, and 112 (male = 60, female = 52) students watched the instructor with nonexpressive nonverbal behavior video lecture. The procedure was as follows: firstly, participants filled in demographic information and took the prior knowledge test. Secondly, they viewed one of two system-paced video lecture. Thirdly, they filled out the learning experience questionnaire and the learning satisfaction questionnaire. Finally, they took the learning performance test. It tooks approximately 30 min to complete the experiment.

### Results

Four independent sample *t*-tests were conducted with experiment conditions (expressive vs. nonexpressive) as the between-subject factor, prior knowledge, and the following three measures as dependent measures: learning performance, learning experience, and learning satisfaction. There was no significant difference in prior knowledge between the two groups (*p* = 0.053>0.05, Cohen’s *d* = 0.054), prior knowledge score for expressive condition *M* = 3.47, SD = 1.003 and for nonexpressive condition *M* = 3.52, SD = 0.849. Descriptive statistics for all dependent variables are shown in Table 2.

**Table 2:** The descriptive statistics for dependent variables in experiment 1.

Dependent variable	Expressive (*N* = 115)		Nonexpressive (*N* = 112)		*t*	*d*	Sig.
	*M*	SD	*M*	SD			
Learning performance	12.60	3.612	16.04	2.421	−8.459	1.118	<0.001^**^ (2-tailed)
Learning experience	40.16	15.119	40.16	13.174	−0.002	0	0.499 (1-tailed)
Learning satisfaction	12.04	2.252	11.03	2.183	3.171	0.455	0.001^*^ (1-tailed)

* *p* < 0.05;

** *p* < 0.001.

#### Learning performance

The independent sample *t*-test showed that there was a significant difference in learning performance between the two groups (*t* = −8.459, *p* < 0.001). Students in the nonexpressive condition performed much better than students in the expressive condition (Cohen’s *d* = 1.118).

#### Learning experience

The independent sample *t*-test showed that there was no significant difference in learning experience (*t* = −0.002 *p* = 0.499) between the two groups. To compare differences, we performed 9 independent sample *t*-tests with each sub-item in the learning experience as the dependent variable and experiment conditions (expressive vs. nonexpressive) as the between-subject factor. Descriptive statistics are shown in Table 3. A significant difference in perceived difficulty between the two groups (*t* = 0.971, *p* = 0.010) was found. The mean values in brackets in the row of perceived task difficulty in Table 3 are the results of reverse scoring, which was used to calculate the overall learning experience. Students in expressive condition thought the content was less difficult. Additionally, instructors with expressive nonverbal cues could be significantly more effective in facilitating participants’ enjoyment (*t* = 3.275, *p* = 0.007 < 0.05) and desire to learn more knowledge about the material (*t* = 1.552, *p* = 0.012 < 0.05). The calculation results of the effect size indicator Cohen’s d show that the three items E1, E2, and E5 all were low effects[d(E1) = 0.136, d(E2) = 0.455 d(E5) = 0.219]. No other significant differences were found (*p* > 0.05) on the left 6 sub-items. Overall, our results partly support hypothesis 1.

**Table 3:** Results of independent sample *t*-test of learning experience sub-items in experiment 1.

Source of variation	Expressive condition (*N* = 115)		Nonexpressive condition (*N* = 112)		*t*	*d*	Sig. (1-tailed)
	*M*	SD	*M*	SD			
E1. I felt that the subject matter was difficult.	1.93(5.07)	1.464	2.15(4.85)	1.750	0.971	0.136	0.010^*^
E2. I enjoyed learning this way.	5.50	1.360	4.80	1.698	3.275	0.455	0.007^*^
E3. I would like to learn this way in the future.	4.56	1.791	4.40	1.814	0.669	0.089	0.494
E4. I feel like I have a good understanding of the material.	4.91	1.621	4.99	1.683	−0.346	−0.048	0.499
E5. After this lesson, I would be interested in learning more about the material.	5.83	1.273	5.51	1.626	1.552	0.219	0.012^*^
E6. I found the lesson to be useful to me.	5.25	1.569	5.04	1.695	0.934	0.129	0.168
E7. I felt like the instructor was working with me to help me understand the material.	4.63	1.654	4.91	1.713	−1.177	−0.166	0.474
E8. I found the instructor’s teaching style engaging.	4.39	1.836	4.05	1.811	1.318	0.186	0.211
E9. I felt motivated to try to understand the material.	5.11	1.581	4.73	1.789	1.610	0.225	0.055

* *p* < 0.05.

#### Learning satisfaction

Students in the expressive group were more satisfied than those in the nonexpressive group. There was a significant difference in learning satisfaction between the two groups (*t* = 3.171, *p* = 0.001, Cohen’s *d* = 0.455). Overall, the result supported hypothesis 2.

### Discussion

Students performed better in the learning performance test when the instructor with nonexpressive nonverbal cues. This was an interesting and counterintuitive finding. Intuitively, the instructor with expressive nonverbal behavior worked “harder” and students “should” learn more from her. However, it was not the case. Previous studies (McKinney and Larkins, 1982) have shown that students had the highest achievement scores when they were in the medium enthusiastic-teacher condition rather than in the high enthusiastic-teacher condition. Two reasons may contribute to the results. Firstly, when instructors with expressive nonverbal behavior, they attract more attention from students, resulting in reduced student attention to learning materials. According to Mayer’s selecting-organizing-integrating theory of active learning, attention, which occurs in the stage of information selection, is the basis of information organization and integration (Mayer and Moreno, 2003). When students spent less time paying attention to materials, it will harm the organization and integration of learning material information. Secondly, elementary students with limited working memory. Instructors’ nonverbal information occupies learners’ limited working memory, which affects the processing of learning material. The simultaneous processing of nonverbal information and learning material information and the integration of the two put forward high requirements for learners’ cognitive processing.

Although there was no significant difference in the overall learning experience between the two groups, there were significant differences in three sub-items. Students who watched the instructor with expressive nonverbal behavior found the content easier (e.g., “I felt that the subject matter was difficult,” the less the score, the easier), more preferred the instructional video (e.g., “I enjoyed learning this way”), and were more interested in learning (e.g., “After this lesson, I would be interested in learning more about the material”). It was consistent with a previous study. Lawson et al. (2021b) proved that positive instructors promoted the enjoyment of the lesson. This can be explained from both emotional contagion and emotional response theory. From the emotional contagion perspective, the instructor’s smiling expression and changing intonation conveyed positive emotional information to students, making students’ emotions more positive. From the emotional response perspective, due to the beneficial social cues provided by the expressive instructor, the connection between students and the instructional video was strengthened, leading to a higher emotional evaluation of the video. The reason for interest difference may be that students are more interested in the content they are not familiar with and have not mastered, which can be proved by students’ learning performance scores. In addition, from the perspective of emotion, students are easy to be interested in the content they like. As we already know, students in the expressive condition enjoyed the course more, which may have led to a higher interest in future learning. Concerning learning satisfaction, instructors’ expressive nonverbal behavior enhanced students’ satisfaction which is consistent with the previous study (Wang et al., 2019). This may be because the positive nonverbal cues of instructors won the favor of students, which can be proved by the degree of students’ enjoyment of the course.

In sum, instructors’ expressive nonverbal behavior had an opposite effect on students’ learning performance and affective experience. Expressive nonverbal behavior promoted students’ affective experience but hindered their learning performance. The instructor with nonexpressive nonverbal behavior was more conducive to elementary students’ performance in the post-test. Concerning affective experience, the instructor with expressive nonverbal behavior improved students’ enjoyment and learning interests. What’s more, students were more satisfied in the expressive condition than those in the nonexpressive condition.

## Experiment 2

### Aim and hypothesis

The purpose of experiment 2 was to verify the conclusions of experiment 1, so we chose a different sample to conduct the experiment. Based on the findings of experiment 1, the following hypotheses were formulated:

*H3*: Compared with the expressive condition, students who learn from the instructor with nonexpressive nonverbal behavior will perform better in learning performance test.

*H4*: Compared with the nonexpressive condition, students who learn from the instructor with expressive nonverbal behavior will have a better learning experience.

*H5*: Compared with the nonexpressive condition, students who learn from the instructor with expressive nonverbal behavior will report higher learning satisfaction.

### Method

#### Participants and design

Participants in the present study were students from an urban elementary school located in Wuhan, China. A total of 175 students in sixth grade responded to the survey. The sample consisted of 87 males and 88 females. All participants were randomly assigned to two groups: 87 students (male = 41, female = 46) watched the video lecture with an expressive instructor and 88 (male = 46, female = 42) students watched video lecture with a nonexpressive instructor. The data were collected through questionnaires that were administered in the school classes by a research assistant and four school instructors. The study protocol was approved by the ethics committee of Central China Normal University.

#### Material

The two versions of the video lectures were the same as experiment 1.

#### Measurements

The pre-test, post-test, and post-questionnaires were the same as in experiment 1.

#### Procedure

The study was carried out in four multimedia classrooms, with four naturally occurring classes. Each condition was randomly assigned to two classes. 87 students (male = 44, female = 43) watched the instructor with expressive nonverbal behavior video lecture, and 86 (male = 42, female = 44) students watched the instructor with nonexpressive nonverbal behavior video lecture. The procedure was as follows: firstly, participants filled in demographic information and took the prior knowledge test. Secondly, they viewed one of two system-paced video lectures. Thirdly, they filled out the learning experience questionnaire and learning satisfaction questionnaire. Finally, they took the post-test. It took approximately 30 min to complete the experiment.

### Results

Four independent sample *t*-tests were conducted with experiment conditions (expressive vs. nonexpressive) as the between-subjects factor, prior knowledge, and the following three measures as dependent measures: learning performance, learning experience, and learning satisfaction. There was no significant difference in the prior knowledge between the two groups (*t* = 1.08, *p* = 0.281 > 0.05). The mean and standard deviation for the expressive condition were *M* = 4.08 and SD = 1.081, and for the nonexpressive condition were *M* = 3.90 and SD = 1.155. Descriptive statistics for all dependent variables are shown in Table 4.

**Table 4:** The descriptive statistics for dependent variables in experiment 2.

Dependent variable	Expressive condition		Nonexpressive condition		*t*	*d*	Sig. (1-tailed)
	*M*	SD	*M*	SD			
Learning performance	17.51	3.121	17.57	2.986	−0.135	−0.020	0.447
Learning experience	48.39	10.01	43.79	12.33	2.658	0.410	0.005^*^
Learning satisfaction	11.33	2.38	10.57	3.03	1.774	0.279	0.039^*^

* *p* < 0.05.

#### Learning performance

We did not find a significant difference in learning performance between the two groups (*t* = −0.135, *p* = 0.447 > 0.05). The nonexpressive group (*M* = 17.57, SD = 2.986) performed slightly but not significantly (*p* = 0.447 < 0.05) higher than the expressive group (*M* = 17.51, SD = 3.121). Hypothesis 3 was violated.

#### Learning experience

The independent sample *t*-test result showed that there was a significant difference in learning experience between the two groups (*t* = 2.658, *p* = 0.005 < 0.05, Cohen’s *d* = 0.410). To compare differences, we performed 9 independent sample *t*-tests with each sub-item in the learning experience as the dependent variable and experiment conditions (expressive vs. nonexpressive) as the grouping variable. Descriptive statistics are shown in Table 5. Specifically, there was significant difference on perceived difficulty (*t* = 2.286, *p* = 0.003 < 0.050, Cohen’s *d* = 0.432), learning interest (*t* = 2.342, *p* = 0.010 < 0.050, Cohen’s *d* = 0.359), and usefulness (*t* = 1.864, *p* = 0.032 < 0.050, Cohen’s *d* = 0.286). It was consistent with experiment 1 that students in the expressive condition perceived the content as less difficult. And they became more interested in the learning topic. The result supported hypothesis 4.

**Table 5:** Results of independent sample *t*-test of learning experience sub-items in experiment 2.

Source of variation	Expressive condition		Non-expressive condition		*t*	*d*	Sig. (1-tailed)
	*M*	SD	*M*	SD			
E1. I felt that the subject matter was difficult.	1.15 (5.85)	1.393	1.81 (5.19)	1.653	2.286	0.432	0.003^*^
E2. I enjoyed learning this way.	5.40	1.466	4.99	1.801	1.630	0.250	0.053
E3. I would like to learn this way in the future.	4.83	1.543	4.58	1.818	0.963	0.148	0.169
E4. I feel like I have a good understanding of the material.	5.84	4.647	5.01	1.719	1.561	0.237	0.060
E5. After this lesson, I would be interested in learning more about the material.	5.76	1.478	5.18	1.742	2.342	0.359	0.010^*^
E6. I found the lesson to be useful to me.	5.33	1.459	4.86	1.814	1.864	0.286	0.032^*^
E7. I felt like the instructor was working with me to help me understand the material.	5.41	1.560	5.01	1.771	1.562	0.240	0.060
E8. I found the instructor’s teaching style engaging.	4.47	1.868	4.14	1.955	1.149	0.173	0.126
E9. I felt motivated to try to understand the material.	5.08	1.723	4.75	1.827	1.230	0.186	0.110

* *p* < 0.05.

#### Learning satisfaction

There was a significant difference in learning satisfaction between the two groups (*t* = 1.774, *p* = 0.039 < 0.050, Cohen’s *d* = 0.279). Descriptive statistics are shown in Table 4. It was found that students in expressive condition were more satisfied than students in nonexpressive condition. Hypothesis 5 was supported.

### Discussion

Experiment 2 aimed to find out if students who watched the expressive video had a better affective experience but worse learning performance than those who watched the nonexpressive video. In experiment 2, the same procedure was conducted but a sample from a different city was selected. Results showed that students who watched the expressive videos did report a higher learning experience, but there was no significant difference in learning performance between the two groups. By comparing the pretest scores of students in the two experiments in detail, we found that the pre-test scores of students in experiment 2 were higher than those in experiment 1(*M* = 3.50 in experiment 1, *M* = 3.99 in experiment 2). In experiment 2, students in urban areas scored slightly better on the prior knowledge test than those in rural areas. For participants in experiment 2, instructors’ nonexpressive nonverbal behavior had no significant effect on their learning performance may because they had relatively higher prior knowledge. The phenomenon that approaches and instructional design that work well for individuals with low knowledge experience may not work well for individuals with high knowledge experience is called the expertise reversal effect (Kalyuga et al., 2003). In the field of multimedia learning, learners’ previous knowledge is an important factor affecting learning results (Kalyuga, 2007).

Students in experiment 2 reported significant differences in learning experience, while students in experiment 1 did not report significant differences in the overall learning experience. It is possible that there is a correlation between the learning experience and the learning performance: low learning performance put a negative effect on the learning experience, while high learning performance put a positive effect on the learning experience. In experiment 1, the learning performance of students in the expressive group was significantly lower than those of the nonexpressive condition, which offset the good learning experience of instructors’ expressive nonverbal behavior. The opposite was true for the nonexpressive condition. Thus, there was no significant difference in the learning experience of the two groups. There’s another possibility, rural students with low prior knowledge find the learning content difficult (*M* = 1.84 in experiment 1, *M* = 1.48 in experiment 2, the less scores, the easier). To understand the learning content, they pay more attention to the learning material and do not pay much attention to the expression of instructors’ nonverbal behavior. Students with high prior knowledge have less pressure to master the learning content and pay more attention to the instructors’ nonverbal behavior. Therefore, the learning experience was not significantly different in Experiment 1 and significantly different in Experiment 2.

## Conclusion

This study conducted two rounds of experiments to explore the effects of instructors’ nonverbal expressiveness in video lectures on primary school students’ learning performance and affective experience (including learning experience, and learning satisfaction). Counterintuitively, both experiments showed that instructors’ expressive nonverbal behavior decreased students’ perception of the learning difficulty, that is, students who watched the expressive nonverbal behavior video thought the teaching content was less difficult than those who watched the nonexpressive video. Interestingly, we also found an expertise reversal effect, that is, methods that work for learners with low prior knowledge do not work for those with high prior knowledge. Students from urban areas in experiment 2 performed better on prior knowledge test than students from rural areas in experiment 1. As a result, unlike in experiment 1, nonexpressive instructors promoted students’ learning performance, in experiment 2, no significant difference in learning performance was found. In terms of learning experience and satisfaction, both experiments showed that instructors’ expressive nonverbal behavior improved students’ learning experience (for experiment 1, it improved the learning experience of individual sub-items, and for experiment 2, it improved the overall learning experience) and satisfaction.

## Contribution and implication

This paper adds important information to the multimedia learning literature regarding instructors’ nonverbal expressiveness on elementary students’ learning performance and affective experience. Instructors’ expressive nonverbal behavior enhances students’ affective experience but hinders students’ learning performance when students’ prior knowledge is low. Besides, it further supports the limited-capacity assumption that each channel in the human information processing system has a limited capacity. Instructors’ expressive nonverbal cues may increase the amount of information processing, which led to the decline of students’ learning performance when students with low prior knowledge.

The implications of this research for teaching are that instruction should be based on learners’ prior knowledge. Facing learners with different prior knowledge, instructors’ expressive nonverbal behavior will have different effects. For learners with low prior knowledge, instructors’ expressive nonverbal behavior hindered learners’ learning performance. While for learners with high prior knowledge, instructors’ expressive nonverbal behavior did not hinder learners’ learning performance. Considering the long-term development of learners, we do not advise instructors to adopt nonexpressive nonverbal behavior even facing learners with low prior knowledge. Because compared to expressive instructors, nonexpressive instructors were less efficient in improving learners’ affective experience. For learners with low prior knowledge, instructors should adopt expressive nonverbal behavior, but reduce the difficulty of learning content or provide learners with more scaffolds, so as to help students achieve good learning performance, meanwhile obtaining a good affective experience and maintaining interest in learning.

## Limitations and future research

Despite the meaningful and interesting findings, this study has three aspects of limitations that need to be further considered. First, we did not use eye trackers to track students’ attention due to the large sample size and equipment shortage. This makes it lack evidence for us to compare whether there are differences in learners’ attention distribution on instructors and learning content. In future research, we recommend using eye trackers to record the attention distribution of learners with different prior knowledge when watching video lectures with different nonverbal expressiveness instructors. Second, we did not measure learners’ perceptions of the instructor’s professional level and emotional state. Instructors’ nonverbal behavior is more than cognitive information for learners, it is essential emotional information and social cues. Learners might perform differently when learning from a perceived expert than a perceived novice (Beege et al., 2019). What’s more, when learners perceive the instructor’s emotion as positive, learners may be more willing to engage in learning activities. The third limitation of the study is that the findings are not generalizable across cultures. This study was conducted in China, and its conclusions are more applicable to Chinese students. We know that nonverbal expressiveness is closely related to culture. What is considered expressive nonverbal behavior in the context of Chinese culture may not be expressive enough in the context of Western culture. Future research should explore the impact of different levels of nonverbal expressiveness of instructors on young learners in different countries and cultural backgrounds.

## Data availability statement

The original contributions presented in the study are included in the article/supplementary material, further inquiries can be directed to the corresponding author.

## Ethics statement

The studies involving human participants were reviewed and approved by the Central China Normal University. Written informed consent for participation was not required for this study in accordance with the national legislation and the institutional requirements. Written informed consent was obtained from the individual(s) for the publication of any identifiable images or data included in this article.

## Author contributions

MW and ZC conceived the study. ZC contributed to the supervision. MW, YS, and CX conducted the experiment and collected the data. YS and ZW analyzed and interpreted the data. MW contributed to the writing of the manuscript. All authors contributed to the article and approved the submitted version.

## Funding

This work was supported by the National Natural Science Foundation of China (grant no. 62077022), the Research Project of National Collaborative Innovation Experimental Base for Teacher Development of Central China Normal University (grant no. CCNUTEIII 2021-21), and the Central China Normal University Graduate Education Innovation Funding Project (grant no. 2022CXZZ032).

## Conflict of interest

The authors declare that the research was conducted in the absence of any commercial or financial relationships that could be construed as a potential conflict of interest.

The reviewer JH declared a shared affiliation with the authors to the handling editor at the time of review.

## Publisher’s note

All claims expressed in this article are solely those of the authors and do not necessarily represent those of their affiliated organizations, or those of the publisher, the editors and the reviewers. Any product that may be evaluated in this article, or claim that may be made by its manufacturer, is not guaranteed or endorsed by the publisher.

## Footnotes

^1^Kaufman, P. (1975). *The effects of nonverbal behavior on performance and attitudes in a college classroom*. Unpublished doctoral dissertation, Oklahoma State University.
